# Automatic Crop Canopy Temperature Measurement Using a Low-Cost Image-Based Thermal Sensor: Application in a Pomegranate Orchard under a Permanent Shade Net House

**DOI:** 10.3390/s23062915

**Published:** 2023-03-08

**Authors:** Jaime Giménez-Gallego, Juan D. González-Teruel, Pedro J. Blaya-Ros, Ana B. Toledo-Moreo, Rafael Domingo-Miguel, Roque Torres-Sánchez

**Affiliations:** 1 Department of Automation Engineering, Electrical Engineering and Electronic Technology, Technical University of Cartagena, Campus Muralla del Mar s/n, E-30202 Cartagena, Spain; 2 Department of Agronomic Engineering, Technical University of Cartagena, Campus Paseo Alfonso XIII 48, E-30203 Cartagena, Spain

**Keywords:** precision agriculture, deficit irrigation, crop water stress, Infrared Radiometer, thermography, image segmentation

## Abstract

Water scarcity in arid and semi-arid areas has led to the development of regulated deficit irrigation (RDI) strategies on most species of fruit trees in order to improve water productivity. For a successful implementation, these strategies require continuous feedback of the soil and crop water status. This feedback is provided by physical indicators from the soil–plant–atmosphere continuum, as is the case of the crop canopy temperature, which can be used for the indirect estimation of crop water stress. Infrared Radiometers (IRs) are considered as the reference tool for temperature-based water status monitoring in crops. Alternatively, in this paper, we assess the performance of a low-cost thermal sensor based on thermographic imaging technology for the same purpose. The thermal sensor was tested in field conditions by performing continuous measurements on pomegranate trees (*Punica granatum* L. ‘Wonderful’) and was compared with a commercial IR. A strong correlation (R^2^ = 0.976) between the two sensors was obtained, demonstrating the suitability of the experimental thermal sensor to monitor the crop canopy temperature for irrigation management.

## 1. Introduction

Agriculture in arid and semi-arid regions is mainly constrained by water scarcity. Global population growth and climate change will accentuate the need for a more efficient use of water resources [1]. Consequently, the objective of optimizing water consumption for irrigated agriculture has been widely addressed in the scientific literature, leading to the proposal of alternative irrigation techniques such as RDI strategies [1,2,3,4,5,6,7,8]. These strategies are based on the reduction in irrigation endowment during non-critical periods for the crop. However, the application of deficit irrigation generates water stress in the plant, which may have a detrimental effect on the quantity and quality of the yield, as well as on the plant’s health [9]. To avoid these negative effects, it is necessary to supervise the crop water status by means of physiological indicators [2,3,4,10,11,12]. The midday stem water potential (Ψ_stem_) is considered as the reference indicator of the crop water status [13]. Nonetheless, its measurement procedure is destructive, non-automatable, and time-consuming [5,14], limiting its use for continuous monitoring and large plantations. Alternatively, other soil and atmospheric variables are measured, which allow for an indirect, remote, and continuous estimation of the crop water status [8,15,16,17]. One of the variables used for this purpose is the crop canopy temperature (T_C_), which depends on crop transpiration rates and therefore, on the crop water status. As a thermal regulation mechanism, plants control evapotranspiration by stomatal aperture [16,18,19,20,21]. Water stress situations cause the restriction of the stomatal aperture, avoiding water loss, and leading to a leaf temperature increase [20]. It is possible to measure stomatal conductance directly by using leaf porometers or a portable gas exchange system [16], which determine stomatal closure, but their practicality is limited due to automation difficulties and the time required for adequate sampling [20]. Instead, the difference between T_C_ and air (T_A_) temperatures, and the Crop Water Stress Index (CWSI) [19,22] are widely and successfully used as crop water stress indicators [5,23,24,25,26,27].

The most popular option for the remote and robust measurement of T_C_ is the deployment of IRs. However, their main drawback is that not only is the radiation emitted and reflected by the surface of interest integrated in the temperature measurement, but also from all the surfaces within the field of view of the sensor. Hence, it is impossible to exclusively discriminate the temperature of the crop canopy without a thorough orientation of the sensor. To this end, there are tools available to assist in the installation process [28], even though, for continuous monitoring, the likelihood of the sensor and target leaves changing their position over time is determinant. Alternatively to IRs, thermography is widely used in agriculture [23,24,25,26,29,30,31,32], either handheld [5,24,33], stationary on-site [34,35], land vehicle-attached [36,37], airborne vehicle-attached [27,30,38,39,40], or from satellites [41]. In case of long and medium distance remote measurements, certain limitations and drawbacks must be considered. Satellite measurements have a low spatial and temporal resolution; drones have a limited flight autonomy and expert technicians are required to operate them, which makes it an expensive and restrictive option. Moreover, they require radiometric calibration and atmospheric and geometric corrections for a suitable operation in photogrammetric applications [30]. 

Thermography temperature measurement allows filtering out those regions of the thermal image that do not correspond to the crop canopy. This is crucial when obtaining CWSI, since considering the temperature of other elements, such as the soil or the branches, would lead to misleading results [22,23,27,32]. For automating the process of extraction of the Region Of Interest (ROI), image segmentation techniques are usually implemented. Nevertheless, performing image processing for segmentation on a thermal image involves problems associated with resolution, requiring the use of high quality and costly thermal devices, limiting their use in agriculture as an automatable sensor. Furthermore, even if high-resolution thermal cameras are used, a temperature contrast between the crop canopy and the rest of the elements present in the frame is needed to conduct the segmentation process; although, if the crop is under severe water stress conditions, the ROI can be confused with the rest of the image [42]. Consequently, the use of an auxiliary visible image for a finer segmentation at a lower cost was proposed [24,37]. 

A wide variety of classical computer vision methods have been applied in leaf-based visible image segmentation [43]. In recent studies, techniques based on artificial intelligence have been developed [42,44,45,46]. These are state-of-the-art approaches, since they have demonstrated superior performance and do not require feature engineering [47]. Subsequently, once the visible image is segmented, the obtained mask is applied to the temperature matrix. An additional advantage of processing the visible image is that it can be used to extract other agronomic parameters of interest. For instance, sunlit and shaded leaves could be segmented, which is relevant for the determination of the crop water status [27]. The crop’s phenotype could also be identified by using image classification models [48]. Thus, an image segmentation model could be automatically selected for case-specific water stress measurements. In addition, harvest-related indicators such as fruit count, size, shape, or color can be determined [49,50,51,52,53,54]. These variables allow for yield, quality, and crop phenological stage predictions to be made [49,55,56,57].

Few low-cost solutions have been developed to measure T_C_ by combining thermal and visible imagery in order to provide information on the crop water status [5,24,33,58,59,60]. One of the preferable options in the literature is the FLIR One sensor from FLIR^®^ Systems (Wilsonville, OR, USA) [24,33,60]. Nevertheless, this sensor has to be attached to a smartphone and user operation is required, so a continuous autonomous measurement is not feasible. Osroosh et al. [37] developed a device for automatic T_C_ measurement based on RGB threshold image processing. However, this segmentation method is highly sensitive to the lighting conditions, image background, and type of crop. Moreover, the device performance was not compared with that of a reference instrument under field conditions, which was potentially affected by the environmental factors [61]. Alternatively, a similar system that based the segmentation on more sophisticated machine learning and deep learning models [44] was proposed [61]. The experimental device was compared with a commercial thermal camera, whose images were manually processed to determine the ROI, obtaining a correlation of up to R^2^ = 0.75 [61]. However, the comparative study was carried out on a small scale and on young trees with a low leaf area index. For these reasons, the aim of this study is to further explore the performance of this experimental device under open-field conditions. To do so, we installed it in a cover-cropped pomegranate orchard together with a commercial IR so that it could be compared with a reference system under similar environmental conditions.

## 2. Materials and Methods

### 2.1. Experimental Site

The trials were performed in a commercial orchard located in Mazarrón, Murcia, Spain (37°37′40.5″ N 1°24′03.9″ W; 17 m above sea level). The measurement days took place in 2021 and 2022 during the fruit growth phase (from June to early September), the ripening phase (from early to late September), and the harvest phase (from late September to late October), which are all crucial phenological stages for pomegranate trees. The plant material consisted of pomegranate trees (*Punica granatum* L. ‘Wonderful’) which were planted at a spacing of 5 m × 3.5 m (571 trees per hectare). A protective shade net was installed over the trees, which reduced the incident photosynthetically active radiation by 30% and the evaporative demand. The pomegranate trees were subjected to two irrigation treatments: (i) a ‘control’ treatment, CTL, irrigated to satisfy the 100% of the crop reference evapotranspiration (ETc) throughout the experimental period, and (ii) a regulated deficit irrigation treatment, DI, irrigated at 50% of ETc during the non-critical periods (flowering, fruit set. and first half of the linear fruit growth; from April until August) and at 100% of ETc during the rest of irrigation season.

### 2.2. Weather Station

Climate variables were obtained by means of a weather station located in the same plot, which was net-covered. Air temperature and relative humidity were measured with an HMP45C sensor (Vaisala Oyj, Helsinki, Finland); the wind speed was measured with an A100R Sensor (Vector Instruments, Rhyl, Wales, UK); and the solar radiation was measured with a CMP3/CMP6/CMP11 pyranometer (Kipp & Zonen B.V., Delft, The Netherlands).

### 2.3. IR Sensor

**Figure 1:**
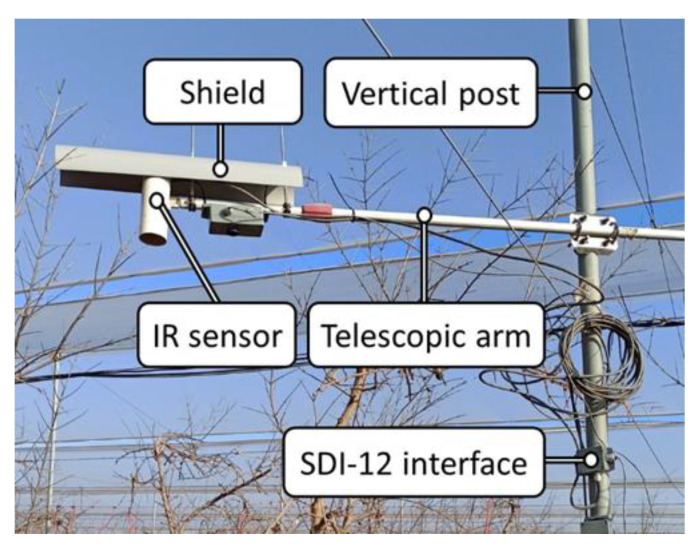
IR set-up detail view.

The commercial IR used for comparison purposes was the model IR120 (Apogee Instruments, Inc., Logan, UT, USA), which, according to the technical specifications, has an effective bandwidth from 8 to 14 μm and an accuracy expected to be ± 0.2 °C. The sensor was located at approximately 0.5 m from the crop canopy pointing downwards. It was mounted on a horizontal telescopic arm attached to one of the vertical metallic posts that support the crop protective net and, to protect the sensor from rain, a semi-cylindrical shield was installed to cover it, as depicted in Figure 1. The assembly allowed the length, the height above the ground, and the angle of the arm with respect to the vertical post to be adjusted. In addition, once the arm position was fixed, it was possible to orient the sensor around the axes of the horizontal plane.

### 2.4. Datalogger

Measurements with the weather station and the IR sensor were performed every 10 min and recorded with a CR1000 (Campbell Scientific Ltd., Logan, UT, USA) datalogger. The datalogger was connected via an Ethernet cable to a router, which allowed the access to the Internet with a mobile connection. The data were sent to a virtual server located at the Technical University of Cartagena, where they were stored in a time series InfluxDB database [62] and displayed on Grafana [63].

A large number of sensors involved in the experimental plot required the use of SDI-12 [64] communication to avoid the installation of additional input/output multiplexers. Thus, in order to communicate the IR sensor, which has an output that is originally voltage-based, with the datalogger via the SDI-12 protocol, a specific interface was developed [65]. The IR temperature was calculated and corrected in the interface with the equations and calibration coefficients provided by the manufacturer [66].

### 2.5. Thermal Sensor

The experimental thermal sensor consisted of a low-cost thermal camera FLIR Radiometric Lepton 3.5 from FLIR^®^ Systems (Wilsonville, OR, USA) with a thermal spectral range from 8 to 14 µm (longwave infrared), an accuracy of ±5 °C, and a resolution of 160 × 120 pixels, mounted on the socket PureThermal 1, FLIR Lepton Smart I/O Module V1.3 de GetLab (Reno, NV, USA); a low-cost RGB camera Raspberry Pi Camera Rev. 1.3 (5 MP); and a processing unit Raspberry Pi 4 model B from Raspberry Pi Foundation (Station Road, Cambridge, UK).

**Figure 2:**
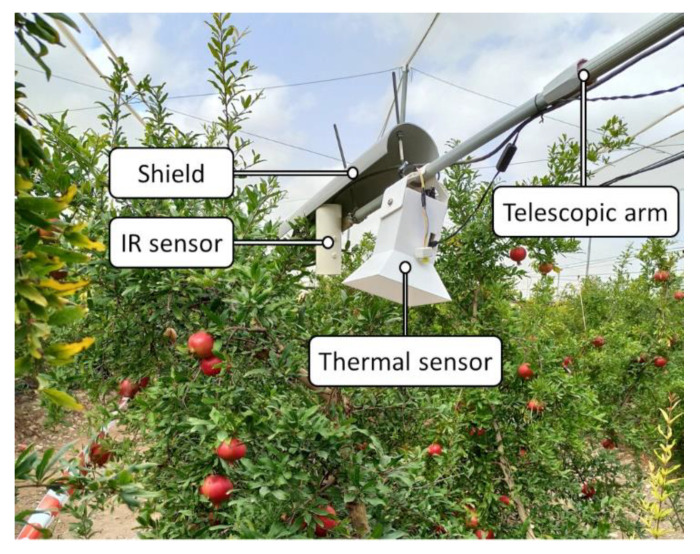
Thermal sensor and IR set-up detail view.

In order to compare the experimental and commercial sensors as accurately as possible, the thermal sensor was installed in the horizontal arm at the minimum possible distance from the IR sensor, as shown in Figure 2, which also allowed both sensors to have similar orientations. Although the canopy region monitored by the two sensors did not fully coincide, they were close to each other.

**Figure 3:**
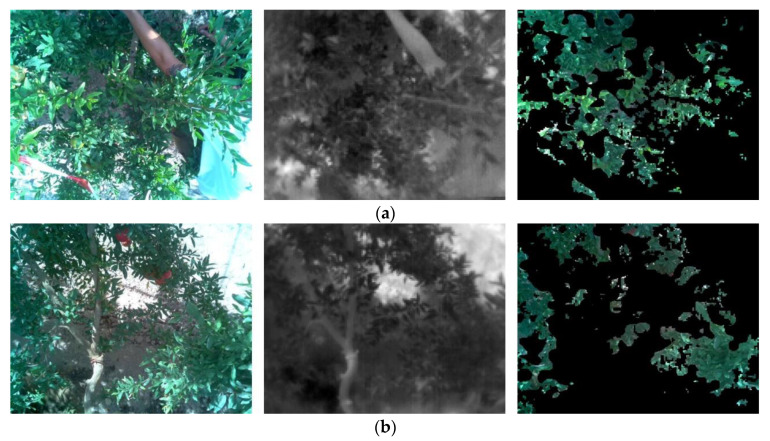
Examples of thermal sensor image processing: (**a**) date: 22 July 2021 (DOY 203) and (**b**) date: 16 September 2022 (DOY 259). From left to right: visible image, thermal image, and mask overlaid on visible image.

**Figure 4:**
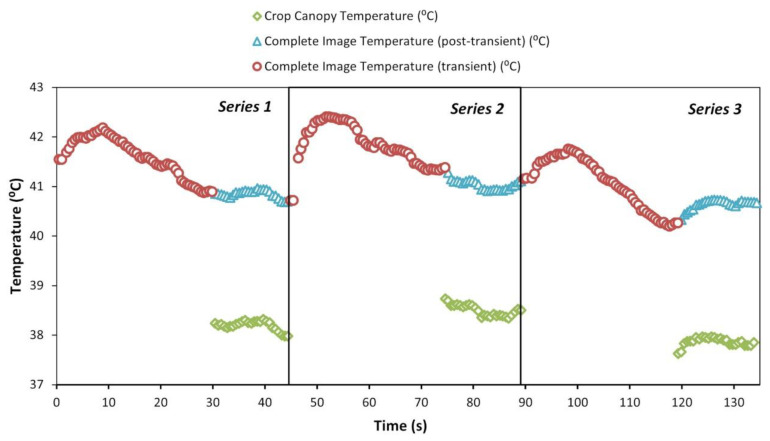
Example of thermal sensor temperature reading: crop canopy (green diamond), complete image post-transient (blue triangle), and complete image transient (red circle). Date: 22 July 2021 (DOY 203). Time: 13:45 (GMT + 2).

As defined in the research by Giménez-Gallego et al. [61], the measurement protocol of the thermal sensor consisted of 3 series of 25 repetitions each, as a lower number of repetitions was demonstrated to be not reliable. Each series started with a Flat Field Correction (FFC) calibration event and a transient period of 30 s to wait for the measurement to stabilize [61]. In every repetition, a visible image of 640 × 480 pixels was captured together with the temperature matrix measured by the thermal camera. Thermographic parameters were set as default, considering emissivity as 1 and reflected temperature as 22 °C. Subsequently, the measurements were processed in a computer (CPU Intel^®^ Core™ i5-8600K, RAM 16 GB, GPU GTX 1070 Ti 8 GB) using Python [67] to perform image segmentation using an artificial intelligence-based model: deep learning model for semantic segmentation with SegNet network architecture [61]. Examples are provided in Figure 3. Once the mask was obtained, it was overlaid on the temperature matrix to filter out the elements that were not part of the ROI. Although both cameras were mounted at minimal distance, to ensure an accurate match between visible image and temperature matrix, a calibration under laboratory conditions was conducted to obtain the intersection region [61]. For the temperatures corresponding to the remaining pixels, the extreme values, i.e., those above the mean plus three times the standard deviation, were also filtered out. The mean, median, and standard deviation were then calculated. The median, which is less sensitive to outliers, was chosen as a representative measure of the repetition. An example of thermal sensor temperature reading is shown in Figure 4. For each series, the mean, median, and standard deviation of all repetitions were computed. As final measurement results, the mean and standard deviation of the medians of the series were calculated. The reading process described above was repeated every 15 min. A total of 120 measurements were performed with the thermal sensor along the experiment, which resulted in an average of 15 measurements per day.

## 3. Results and Discussion

### 3.1. Time Series of Temperature Measurements

**Figure 5:**
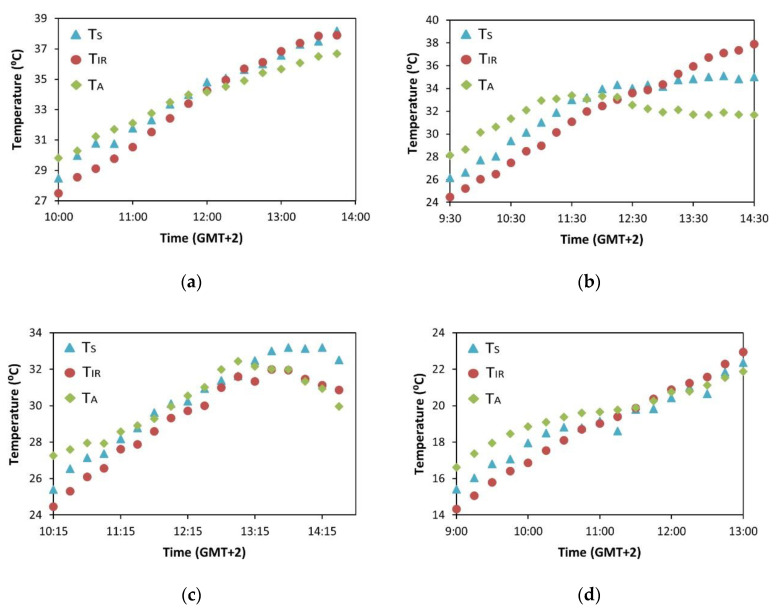
Time series of T_S_ (blue triangle), T_IR_ (red circle), and T_A_ (green diamond) in specific days: (**a**) date: 22 July 2021 (DOY 203), irrigation treatment: CTL; (**b**) date: 22 July 2022 (DOY 203), irrigation treatment: DI; (**c**) date: 16 September 2022 (DOY 259), irrigation treatment: DI; and (**d**) date: 30 September 2022 (DOY 273), irrigation treatment: DI.

The temperature time series were measured every day with the thermal (T_S_) and IR (T_IR_) sensors. Examples of these measurements are presented in Figure 5, together with the T_A_ obtained with the weather station. The thermal sensor had a different sampling period (one sample every 15 min) than the IR one and the weather station (one sample every 10 min). Considering that the experiment started at the same time for all sensors, this resulted in only two synchronized measurements per hour among them. For the rest of the cases, the most frequently sampled measurements, i.e., those from the IR sensor and the weather station, were averaged to approximately match the thermal sensor sampling period.

According to Figure 5, both the thermal and IR sensors showed similar trends. In all cases presented, an intersection of T_S_ and T_IR_ with T_A_ was detected at some point around midday. This effect is explained by the increase in T_C_ caused by stomatal regulation resulting from water stress. Particularly, in Figure 5b,c, the difference between the T_S_ and T_A_ after midday is more noticeable. This might be explained by the fact that these cases correspond to the DI treatment and high air temperatures. Specifically, in Figure 5c, irrigation by the date of the measurement already reached 100% of the ETc (full irrigation), but an incomplete soil water storage can still be assumed after the deficit period. Additionally, in Figure 5c, it can be observed that T_S_ was higher than T_IR_ after midday. In this case, the thermal sensor exhibited a higher Tc than the IR sensor. In Figure 5a,d, the difference between T_S_ and T_A_ after midday is significantly lower. This is attributable to the fact that both treatments were irrigated at 100% of the ETc on the measurement date, and considerable time had elapsed since the water deficit period in the DI treatment, which allowed the water soil reserve and the plant water status to fully recover.

### 3.2. Comparative Analysis between Thermal and IR Sensors

**Figure 6:**
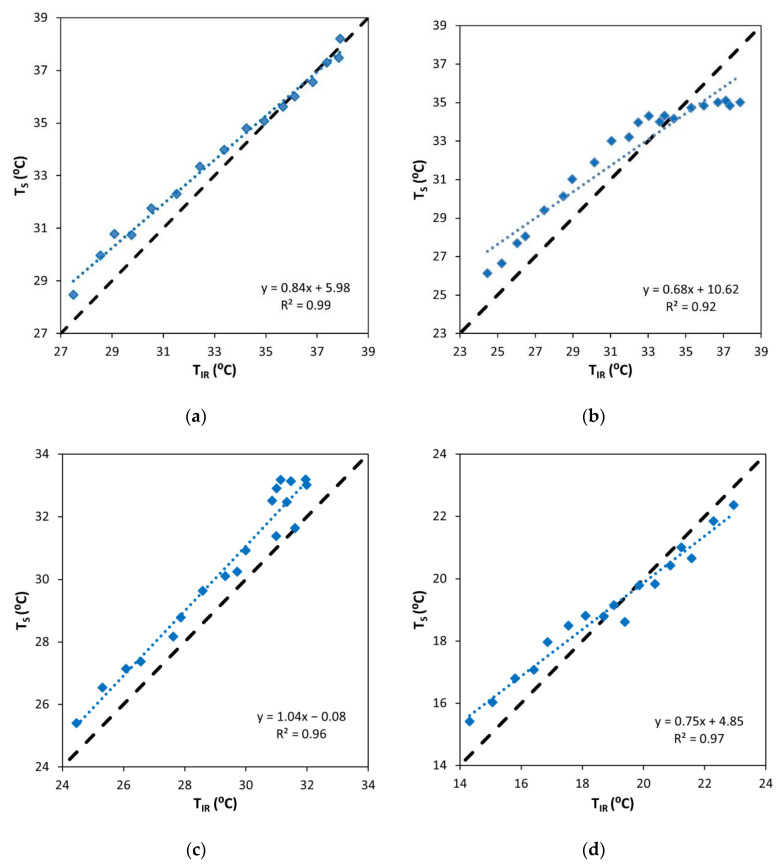
Comparison of the temperature measurements with the thermal (T_S_) and IR (T_IR_) sensors in specific days (blue) and 1:1 line (black): (**a**) date: 22 July 2021 (DOY 203), irrigation treatment: CTL; (**b**) date: 22 July 2022 (DOY 203), irrigation treatment: DI; (**c**) date: 16 September 2022 (DOY 259), irrigation treatment: DI; and (**d**) date: 30 September 2022 (DOY 273), irrigation treatment: DI.

In order to analyze the similitude of the T_C_ measurement between the thermal and IR sensors, the values measured with both sensors are presented in Figure 6 for the same cases included in Figure 5, which are representative of the rest of the measurements obtained in the experiment.

A high correlation between the temperatures measured by the two sensors was observed in general, with the R^2^ values above 0.9 (Figure 6). Nonetheless, in the case of Figure 6b, this correlation was significantly lower. Analyzing this from the perspective of Figure 5, the maximum deviation between the thermal and IR sensors came beyond midday, when the IR sensor measurements deviated from the horizontal asymptote described by the thermal and air temperature sensors. Considering that this effect was not noticed in any other test under similar conditions, its cause might be related to a wrong IR orientation, which could have led to the influence of the ground temperature in the measurement.

**Figure 7:**
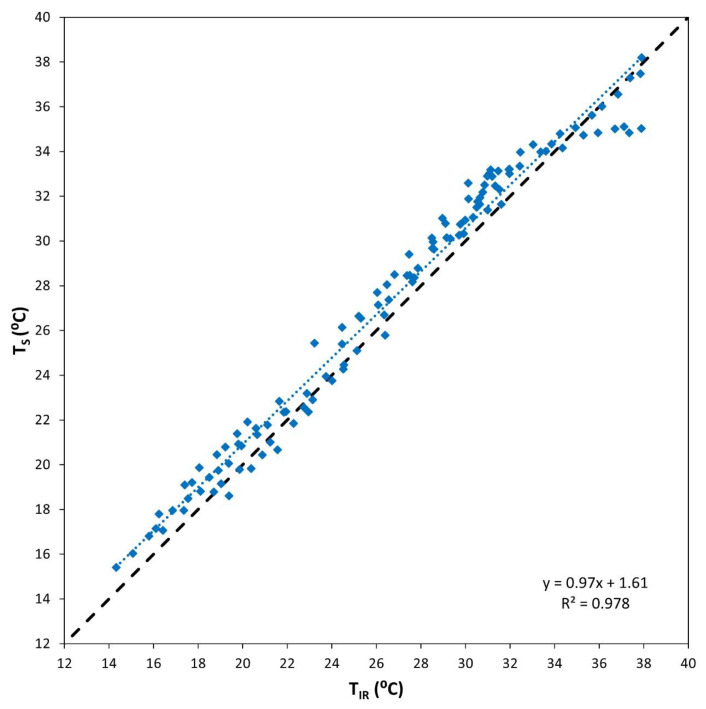
Comparison of all the temperature measurements obtained in the experiment with the thermal (T_S_) and IR (T_IR_) sensors (blue) and 1:1 line (black).

In order to perform a general comparison, all the data obtained along the 8 days of field tests are presented in Figure 7. A strong correlation (R^2^ = 0.978) between the temperatures acquired with the thermal and the IR sensors was achieved, and the maximum deviations corresponding to high temperature samples showed in Figure 5b are discussed above. The slope of the regression line (0.97) was close to a 1:1 line. In the temperature range of the experiment, it was detected that the thermal sensor slightly overestimated the T_C_ with respect to the IR, as the regression line was above the 1:1 line. The mean of the temperature difference between the two sensors resulted in 0.995 °C, whereas the median and standard deviation were 0.960 and 0.623 °C, respectively. The maximum difference was found to be 2.870 °C, corresponding to a measurement made on 2022, DOY 203. In any case, considering the IR sensor as a reference, the maximum deviation incurred by the thermal sensor falls within the accuracy limits provided by the manufacturer of the low-cost thermal camera equipped in the thermal sensor, i.e., ±5 °C.

### 3.3. Analysis of Potential Factors Influencing the Thermal Sensor Performance

The T_C_ measurement may be very sensitive to the variation of environmental parameters (Jones et al., 1997). For this reason, the potential causes of thermal sensor measurement errors were analyzed by examining the influence of the following parameters: wind speed, solar radiation, and air temperature. The relationship of the temperature difference between the thermal and IR sensors (ΔT) with the wind speed was studied for all the data of the experiment. Although high deviations of the thermal sensor temperature were expected at high wind speed values, no evident correlation between the ΔT and wind speed was obtained in the experiments (R^2^ = 0.011). Even so, the wind speed data correspond to single measurements registered every 10 min. Thus, the possible influence of occasional wind gusts, which would have a critical impact on the measurement [34], could not be taken into account. 

With the thermal camera, it is possible to adjust the temperature calculation by tuning certain thermographic parameters. The main ones are emissivity, which is characteristic of the measurement surface, and the reflected temperature, which depends on the external infrared radiation sources existing in the scene and the ambient conditions. These variables were defined as constant values in the experiment, which is appropriate for emissivity, since the target surface is always that of leaves. Nevertheless, significant variations in the ambient conditions may cause an error due to not adjusting the reflected temperature. For example, if the air temperature was significantly different from the reflected temperature set in the thermal imaging camera, the errors would increase. However, no evident correlation between extreme air temperatures and a high ΔT was obtained (R^2^ = 0.014). No correlation was found between the solar radiation and ΔT (R^2^ = 0.011) either. This result was expected, given that when the thermographic measurement is carried out on high emissivity objects, such as those in the field of view of the thermal camera, the reflected radiation does not have a considerable effect [68].

In a previous study [61], the assessment of the same thermal sensor was conducted under different experimental conditions, leading to a lower performance in the estimation of T_C_. The correlation between the temperature obtained with the experimental sensor and the reference was R^2^ = 0.75 in the study by Giménez-Gallego et al. [61], which contrasts with the value R^2^ = 0.978 obtained in this study. Likewise, a higher maximum difference of temperatures between the experimental sensor and the reference was obtained in the study by Giménez-Gallego et al. [61] (4.5 °C vs. 2.87 °C). Since the same experimental thermal sensor was used in both studies, next we will enumerate the different experimental factors that could have potentially led to these considerable differences in the sensor performance.

- Percentage of area covered by leaves in the image of the thermal sensor. In this study, the percentage ranged between 20 and 50%, depending on the orientation set on every day of experimentation, whereas in Giménez-Gallego et al. [61], the range was between 15 and 35%. However, in the case of the latter, additional tests were made by setting the sensor closer to the crop in order to increase the percentage of the canopy covered by the field of view, which did not result in a higher performance.
- Protection against wind. The presence of a cover net above the orchard allowed the sensor to be less exposed to wind in this trial. The neighboring trees and the mounting, as the sensor was enveloped by the perimeter branches of the pomegranate tree, also contributed to this. In contrast, under the experimental setup in the work by Giménez-Gallego et al. [61], the sensor was fully exposed to wind, even so, it was shown that the wind did not affect the temperature measurement considerably, due to the high number of repetitions performed. This allowed us to average and make the T_C_ determination independent of the standard deviation caused by the wind influence.
- Orientation of the sensor. In this paper, the sensor was placed vertically, mounted on the arm pointing downwards, whereas in the work by Giménez-Gallego et al. [61], the sensor was mounted on a horizontal bracket, pointing to the canopy from the side. The horizontal orientation caused the camera’s field of view to encompass the sky and other external elements of the experimental set up, such as roof tiles. These might be more problematic sources of radiation than the ground, which was the background in the case of vertical orientation. Moreover, in the vertical installation, the thermal sensor is less exposed to solar radiation, so the heating of the electronics inside the housing, including the thermal imaging camera, is lower.
- Location of the sensor. In this study, the sensor was placed within the tree canopy, allowing it to be immersed in an atmosphere equal to that of the leaves. This could have made it less sensitive to changes in environmental factors, such as air temperature, relative humidity, and wind, which might have affected the measurement.

Although there is no clear rate of the contribution of each of these factors on the performance of the sensor, it could serve as a guideline to be heeded in the design of new experiments or in the assessment of similar devices.

### 3.4. Optimization of the Thermal Sensor Measurement Procedure

The originally proposed thermal sensor measurement procedure, described in the materials and methods section, involves a large number of repetitions to obtain a single temperature data. This was established in the work by Giménez-Gallego et al. [61] in order to overcome the high standard deviation of the measurements. Nonetheless, the different experimental conditions in this study likely led the standard deviation of the data presented above to be considerably lower than that obtained in the work by Giménez-Gallego et al. [61]. Particularly, the average standard deviation among repetitions of the same series was 0.098 °C, whereas that among series was 0.215 °C. Consequently, a reduction in the number of repetitions performed for every reading is potentially approachable without compromising accuracy. This would decrease the time required for every reading, resulting in lower device energy consumption and lower thermal stress for the electronics. Additionally, the volume of measurement information would be reduced, as would the communication time, the need for storage space, and the processing computational cost of generating results. 

Thus, we reduced the number of repetitions per series from twenty-five to three. Firstly, the three repetitions considered were the first, the intermediate, and the last one. As reported in Table 1, the correlation of the measurements with those from the IR sensor remained high, whereas the temperature difference (ΔT) between both sensors was very similar to that obtained before the optimization. In a second approach, the first three repetitions per series were considered, reducing the measurement time per series from 45 to 32 s. Again, ΔT and the correlation of the measurements between both sensors were practically invariant with respect to the non-optimized measurements. A third alternative approach considered the first three repetitions of a single series. Both the ΔT and the correlation between the sensors were not substantially affected by the optimization, and not only was it possible to reduce the total measurement time to 32 s, but it was also possible to reduce the total number of measurements to three.

## 4. Conclusions

In this article, a low-cost thermal sensor based on thermographic imaging, designed in an earlier work [61], was successfully tested in the field in order to corroborate its performance and usability as a stress monitoring tool in irrigation experiments and its use as an alternative for commercial IRs. Continuous measurements were conducted throughout the morning on different dates and environmental conditions. In addition, potential factors that may influence the sensor’s performance have been discussed. Moreover, an optimized measurement procedure for the thermal sensor was described. With this, a similar performance was achieved at the same time as 96% and 76.3% fewer measurements and measurement times, respectively, were required to obtain a reading.

The experimental thermal sensor was compared with commercial IRs, which are the most commonly used tools for measuring T_C_ under open-field conditions. In accordance with the results, the temperatures measured by both sensors show a strong correlation (R^2^ = 0.976) and an average temperature difference of 1.1 °C. Therefore, regarding temperature measurement, it can be concluded that the thermal sensor is able to monitor T_C_ in a similar way to the commercial IR used as a reference and, thus, can contribute to the knowledge of the crop water status. In terms of material cost, the current state-of-the-art and the evolution of thermographic camera technology lead us to foresee a lower cost and higher resolution in the capture of images, which will provide an economic advantage to the IRs. The complexity of the instrumentation needed to process the signal from some IRs makes them dependent on specific dataloggers or recording systems. This reduces their usage flexibility, as well as entails additional wiring and costs. The field installation of the thermal sensor does not demand a precise and constant orientation over time, which makes the measurement convenient and reliable. However, the hardware robustness and power consumption are factors in favor of IR for long-term and scalable practical use. In our future work, efforts will be made to improve both aspects of the thermal sensor. Furthermore, the possibility of processing the visible image provides great potential for obtaining additional interesting information on the crop.

## Figures and Tables

**Table 1:** Characteristics and performance of the original thermal sensor measurement protocol and alternative optimization approaches.

							ΔT (°C)			
Measurement Approach	Series	Repetitions	Total Measurements	Series Time (s)	Total Measurement Time (s)	R^2^	Mean	Median	Standard Deviation	Maximum
Original	3	25	75	45	135	0.978	0.995	0.96	0.623	2.87
Alternative 1	3	3	9	45	135	0.977	0.998	0.96	0.624	2.93
Alternative 2	3	3 (initial)	9	32	96	0.977	1.061	1.05	0.653	2.81
Alternative 3	1	3 (initial)	3	32	32	0.976	1.103	1.06	0.686	3.05

## Data Availability

Data is unavailable due to privacy restrictions.
